# Indoor nanoscale particulate matter-induced coagulation abnormality based on a human 3D microvascular model on a microfluidic chip

**DOI:** 10.1186/s12951-019-0458-2

**Published:** 2019-02-01

**Authors:** Yan Li, Chuanlin Hu, Pengcheng Wang, Yan Liu, Luyang Wang, Qingmeng Pi, Zhiyong Gong, Xu Yang, Michael Mak, Yang Wu

**Affiliations:** 1 Key Laboratory for Deep Processing of Major Grain and Oil (Wuhan Polytechnic University), Ministry of Education, College of Food Science and Engineering, Wuhan Polytechnic University, Wuhan, 430023 People’s Republic of China; 2 Hubei Key Laboratory for Processing and Transformation of Agricultural Products (Wuhan Polytechnic University), College of Food Science and Engineering, Wuhan Polytechnic University, Wuhan, 430023 People’s Republic of China; 3 Department of Biomedical Engineering, School of Engineering & Applied Science, Yale University, New Haven, 06520 USA; 4 Department of Building Science, Tsinghua University, Beijing, 100084 People’s Republic of China; 5 Harvard-MIT Division of Health Sciences and Technology, Massachusetts Institute of Technology, Cambridge, MA 02139 USA; 6 Hubei Key Laboratory of Genetic Regulation and Integrative Biology, College of Life Sciences, Central China Normal University, Wuhan, 430079 People’s Republic of China; 7 State Key Laboratory of Silicate Materials for Architectures, Wuhan University of Technology, Wuhan, 430070 People’s Republic of China; 8 Department of Plastic and Reconstructive Surgery, Renji Hospital, Shanghai Jiaotong University School of Medicine, Shanghai, 200129 People’s Republic of China

**Keywords:** Indoor particulate matter, Oxidative stress, Inflammation, Promote coagulation, 3D human microvessel

## Abstract

**Background:**

A growing body of evidence shows that indoor concentrations of airborne particles are often higher than is typically encountered outdoors. Since exposure to indoor PM2.5 is thought to be associated with cardiovascular disease, the health impacts of indoor air pollution need to be explored. Based on animal models, ambient particulate matter has been proved to promote coagulation which is very likely involved in the pathogenic development of cardiovascular disease. However, animal models are insufficient to predict what will happen with any certainty in humans. For this reason, the precise pathogenic mechanisms behind the development of cardiovascular disease in humans have not yet been determined.

**Results:**

We generated a 3D functional human microvascular network in a microfluidic device. This model enables human vascular endothelial cells to form tissue-like microvessels that behave very similarly to human blood vessels. The perfusable microvasculature allows the delivery of particles introduced into these generated human-like microvessels to follow the fluid flow. This exposure path effectively simulates the dynamic movement of airborne nanoscale particles (ANPs) within human vessels. In this study, we first identified the existence of ANPs in indoor air pollution. We then showed that ANPs could activate endothelial cells via ROS induced inflammation, and further resulted in abnormal expression of the coagulation factors (TF, TM and t-PA) involved in coagulation cascades. In addition, we found that a protein could cover ANPs, and this biointeraction could interfere with heparan sulfate (HS). Human organotypic 3D microvessel models provide a bridge for how research outcomes can translate to humans.

**Conclusions:**

The 3D human microvessel model was used to determine the physiological responses of human vessels to ANP stimulation. Based on the obtained data, we concluded that ANPs not only disrupts normal coagulation functions, but also act directly on anticoagulant factors in human vessels. These experimental observations provide a potential biological explanation for the epidemiologically established link between ANPs and coagulation abnormality. This organ-on-chip model may provide a bridge from in vitro results to human responses.

## Background

Indoor air pollution in non-occupational settings has attracted increasing public attention more recently [1]. It is because people spend most of their time in different indoor environments. Environmental monitoring data indicated that indoor concentrations of many pollutants (including airborne particles) are often higher than those typically encountered outdoors [2, 3]. Given this, greater attention needs to be paid to the health impacts of indoor air pollution. A growing body of epidemiological data has indicated consistent and coherent associations between particulate matter and an increase in mortality and morbidity [4–6]. For example, numerous epidemiological and clinical studies indicate that ambient particulate matter in air pollution is strongly associated with increased cardiovascular disease [7, 8].

Research has shown that ambient particulate matter-induced coagulation abnormalities are very likely involved in many kinds of cardiovascular disease [9]. The precise biomechanism has not yet been entirely determined. Currently, most studies of indoor particulate air pollution have focused on large particles such as PM2.5 (aerodynamic diameter ≤ 2.5 µm), and PM10 (aerodynamic diameter ≤ 10 µm) [10, 11]. However, among all the noxious pollutants found in indoor air, airborne nanoscale particles (ANP) are the most harmful for human health [12, 13]. ANPs are small enough to invade the smallest airways and to be able to cross some physiological barriers. For example, ANP can more easily cross the lung-blood barrier than the micron-sized airborne particles [14]. ANPs translocate into the bloodstream which is considered to be a significant contributor to the disruption of human vascular function [15, 16]. Therefore, the vascular effects of airborne nanoscale particles should receive a lot more attention.

Once ANPs have entered the bloodstream, they may disrupt endogenous coagulation cascades due to their surface reactivity and specific surface area. For example, nanoparticles could enhance thrombus formation through increased platelet aggregation and procoagulant activity [17]. One accepted explanation is that nanoparticles can aggravate endovascular oxidative stress and inflammation after nanoparticles enter blood vessel [18]. This bioresponse could result in plaque activation which leads to the thrombus formation finally [19]. Also, nanoparticles exhibit extensive surface activity and absorb protein-rich fluids in vivo [20]. This kind of bio-interaction may affect the function of coagulation factors and resulting in coagulation abnormalities. Dobrovolskaia et al. suggested that these procoagulant activities are dependent on particle size and surface charge [21].

Animal models have been widely used in toxicological research. They continue to aid our understanding of airborne particles induced cardiovascular disease [22]. However, the animal model is inaccurate to directly extrapolate discoveries from animals to humans due to species differences [23]. 3D human organotypic cell models have been well developed in recent years. Organotypic cell models could provide primary organ-based physiological information although it is still unable to simulate organ function fully. Moreover, this information is more realistic than results based on 2D human cells. Because cells can regain their physiological form and function when embedded in a three-dimensional (3D) culture environment [24, 25]. For example, 3D cultures allow human vascular endothelial cells in vitro to generate microvascular networks which simulate the physiological function of human blood vessels [26, 27]. This organotypic cell model can more faithfully provide human physiological information than that obtained from cells cultured on a flat surface (2D culture) [28]. Therefore, 3D organotypic cultures provide auxiliary support for animal models in toxicology research. The 3D microvessel model provide us with some human biological information after airborne particles enter the bloodstream.

Fibrin hydrogels are becoming more widely used for tissue engineering. Fibrin hydrogels are a class of biomaterials that have excellent scaffolding potential in 3D cell cultures due to their excellent biocompatibility and appropriate mechanical properties [29]. Fibrin-based scaffolds can represent the 3D space which is available for cells developing into regenerative tissue [30]. The combination of fibrin hydrogel with a microfluidic device has been shown to improve the human physiological relevance of the regeneration of human vessel tissue [31]. This is due to the microfluidic chip provide micro-scale complex structures and well-controlled parameters to mimic the in vivo environment of human vessels [32, 33]. Also, microfluid flow can supply the necessary nutrients, moisture, and oxygen, as well as being able to remove degradation products simultaneously [34, 35]. Microfluid flow has been shown to improve the adhesion, migration, and proliferation of human endothelial cells in 3D scaffolds [36–38].

In this study, the perfusable 3D microvascular networks were constructed in a microfluidic device. In comparison to a 2D cell culture (on a flat dish), the advantage of our 3D model is that the fibrin gel-based scaffolds provide a 3D microenvironment for HUVEC growth. Fibrin hydrogel is a biocompatible synthetic polymeric material. The unique structure and property of fibrin hydrogel can mimic the extracellular matrix (ECM) [39, 40]. HUVECs in this fibrin gel-based scaffold formed capillary-like structures with lumens. We characterized the collected ANPs samples and then analyzed the procoagulant effects on the microvascular networks after ANPs were loaded in regenerated human vessels. We aim to explain the possible mechanism behind ANP promoted abnormalities in human coagulation. Coagulation dysfunction can be indicated by abnormal levels of oxidative stress (ROS, MDA, HO-1), cytokines (NF-κB, IL-1) and coagulation factors (TF, TM, and t-PA). TF is a cell surface glycoprotein responsible for initiating the extrinsic pathway of coagulation. TM and t-PA both play essential roles in the anticoagulant system. The three biofactors are found on endothelial cells, the cells that line the blood vessels. In addition, we hypothesized that ANPs absorb protein to form ANP-protein corona. Based on SEM/EDS, we characterized the bio-interaction of ANPs with plasma protein. Gel electrophoresis was further applied to investigate the potential functional interference as ANPs reacted with HS. The objective of this study was to gain better insight and understanding of indoor ANP-associated human vascular dysfunction.

## Results

### Human microvascular network based on microfluidic 3D HUVEC culture

**Fig. 1:**
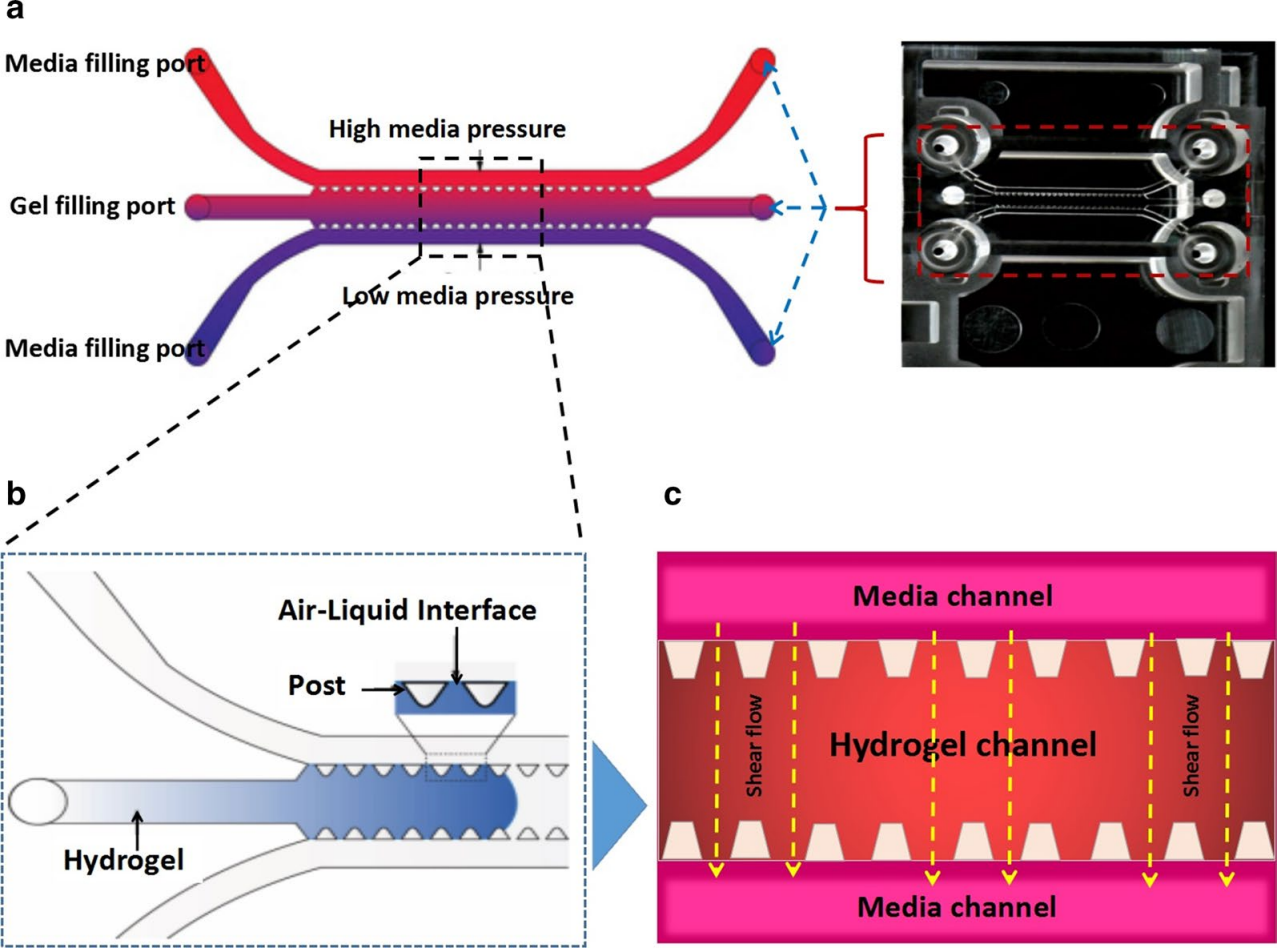
Human 3D microvessel formation based on the combination of fibrin hydrogel with a microfluidic device. **a** Schematic diagram of a microfluidic device. **b** HUVEC/hydrogel mixture injected into the middle channel. **c** Chip generated stress flow to enable the medium to cross the fibrin hydrogel

**Fig. 2:**
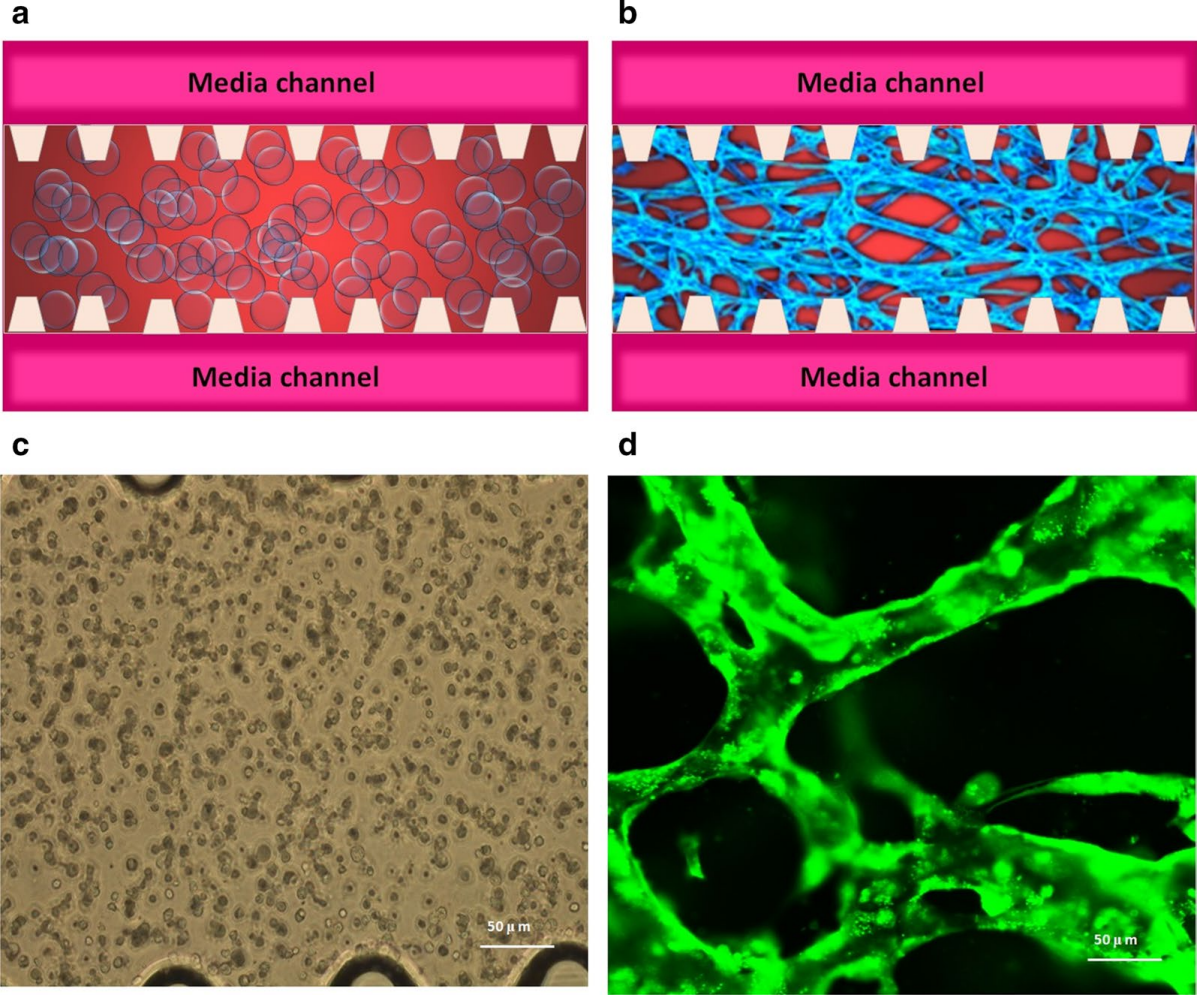
Human microvascular network formation based on microfluidic 3D HUVEC culture. **a** Schematic diagram of HUVECs seeding in the fibrin hydrogel. **b** Schematic diagram of microvascular network formation in the fibrin hydrogel. **c** Microscope image of HUVECs just seeding in fibrin hydrogel. **d** Confocal microscope image of human microvascular networks

**Fig. 3:**
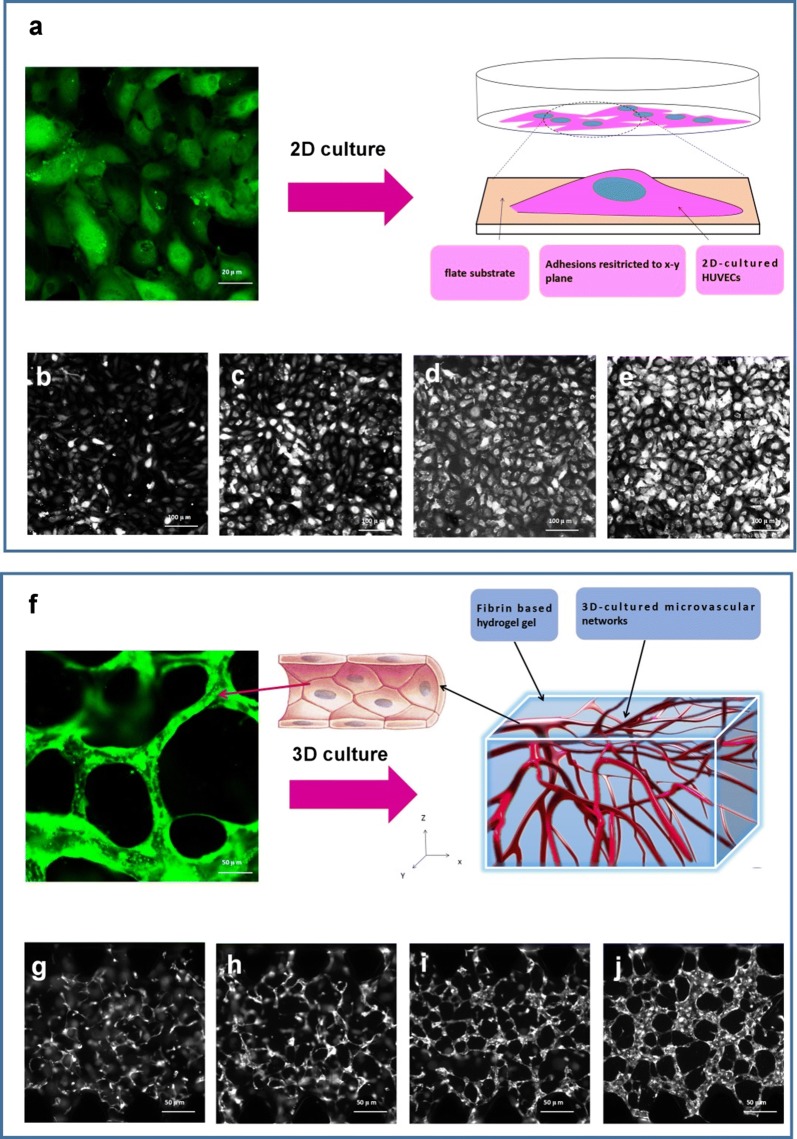
Development progress of human umbilical vein endothelial cells (HUVECs) by 2D culture and 3D culture. **a** 2D HUVEC culture in a disk, **b**–**e** camera image of fluorescent HUVECs developed from day 1 to day 4. **f** 3D HUVEC culture in a chip, **g**–**j** camera image of fluorescent HUVECs developed from day 1 to day 4

In this research, we used a combination of fibrin hydrogel with a microfluidic device to generate 3D human microvascular networks. HUVECs and HLFs were suspended in the scaffold precursor solution of fibrinogen and thrombin. The fibrin precursor was mixed with thrombin to produce fibrin gel. Fibrin gel was applied to generate scaffolds for the 3D organotypic culture of HUVECs. The mixture was delivered into a microfluidic chip (Fig. 1a) to complete the gelation. After gelation, different amounts of HUVEC medium were introduced into two different media channels. A difference in liquid levels in the media channels creates a pressure drop along the gel filling channel (Fig. 1b). The liquid pressure enables the EGM-2 medium to permeate into the fibrin hydrogel (Fig. 1c). With the aid of fluid flow, the microfluidic device offers a dynamic microenvironment for 3D HUVE culture. As shown in Fig. 2a, HUVECs and HLFs were seeded in fibrin hydrogel (Fig. 2c). Fibroblasts are known to secrete growth factors that support endothelial cells forming lumens in three-dimensional co-cultures. In the presence of HLFs, HUVEC spreading is robust and spontaneously form microvascular networks (Fig. 2b) in the microfluidic device (Fig. 2d). In this research, 3D HUVEC microvessel development was monitored via a confocal microscope (Fig. 3f). In the initial stage, HUVECs migrated to form cell–cell adhesions and alignments (Fig. 3g, h). The HUVECs then gradually formed lumens along with cell extension (Fig. 3i). Finally, HUVEC lumens formed microvascular networks (Fig. 3j). For comparison, HUVECs were grown on flat dishes based on 2D cell culture (Fig. 3a). The cells adhered and spread on the plastic surface and formed unnatural cell attachments to the synthetic surface (Fig. 3b–e). All 2D cultured HUVECs generated a single layer of cells but did not form microvascular networks.

### Functional measurement of established 3D human microvessels

**Fig. 4:**
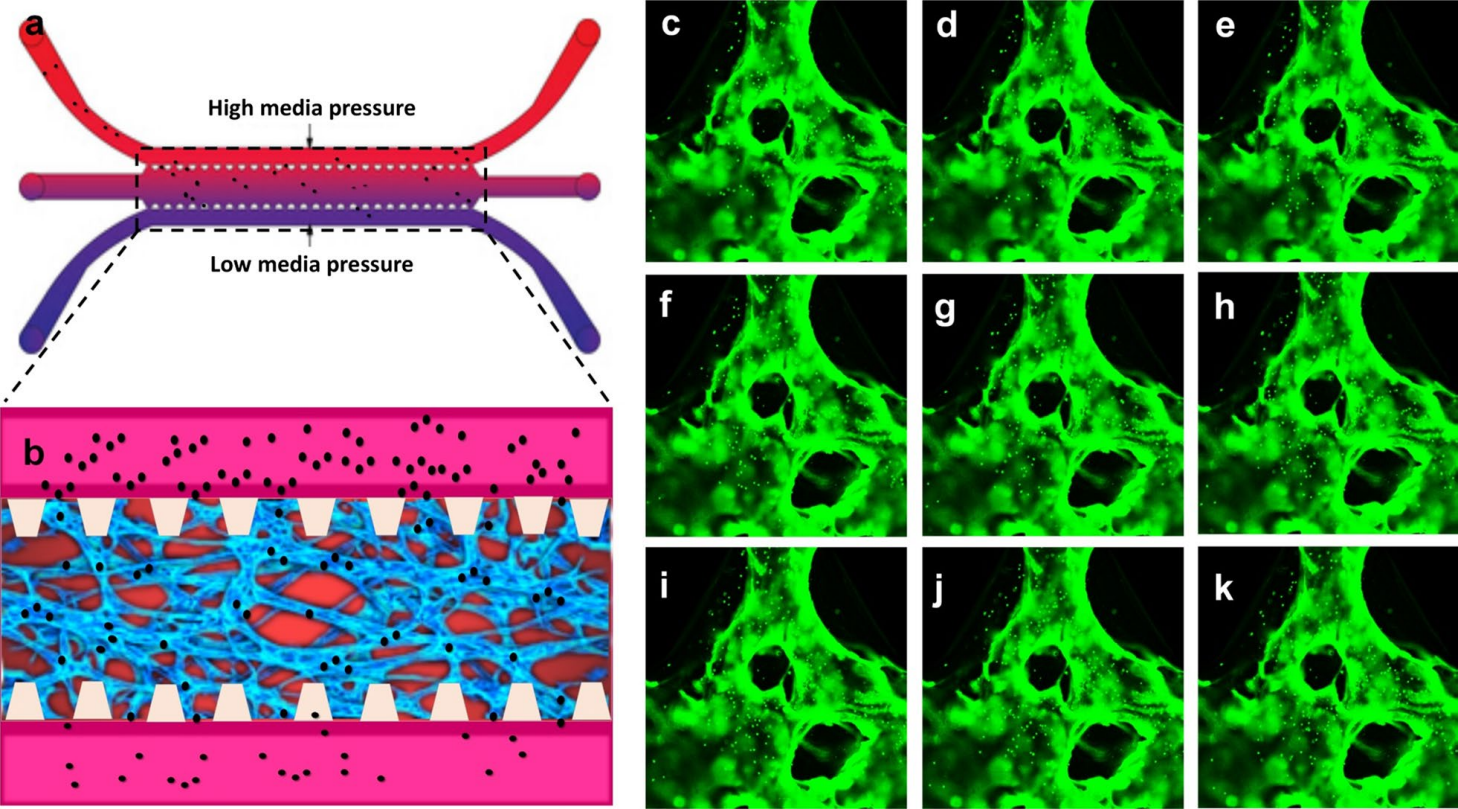
Perfusion measurement of a 3D-cultured human microvascular network. **a** Schematic diagram of loading microparticles in microvascular networks. **b** Chip generated flow to enable microparticles perfused in the microvascular network from the upstream direction. **c**–**k** Confocal microscope images of fluorescent microparticles are moving through the microvascular networks

Based on 3D HUVEC cultures in microfluidic chips, we established 3D human microvascular networks in this research with the aim of mimicking human vessels in a living body. This established 3D vessel model simulated the physiological structure of human vessels. As compared to 2D culture, HUVECs spontaneously formed microvascular networks in this 3D hydrogel microenvironment, and the microscale dimensions of the 3D vessels match the vascular structures inside the human body. In addition, the in vitro human vessel model can still be created to mimic the dynamic function present in real human blood systems. In a living body, the blood vessels have good permeability, which ensures that blood flows through the circulatory system to an organ or tissue. Adequate perfusion of the microvasculature has crucial physiological meaning. In this research, apart from replicating the vessel-like structure, we also measured the perfusion capability for the microvascular networks in the microfluidic device. Fluorescent microparticles (FM) were initially loaded into the microfluidic chip (Fig. 4a). Due to the difference in the liquid levels of two medium channels, the liquid pressure enabled the FM to permeate into the microvascular network (Fig. 4b). The confocal microscope images demonstrated that the FM was migrating through the vessels following the fluid flow. Images illustrate the path of the microparticles in the microvessels. With the aid of fluid flow, the microfluidic device provides the dynamic power that enables the FM to go through the artificial human vessels (Fig. 4c–k). By tracking FM movement, we could evaluate the microvascular network, which exhibited excellent perfusion ability.

### Physicochemical characteristics of ANP

**Fig. 5:**
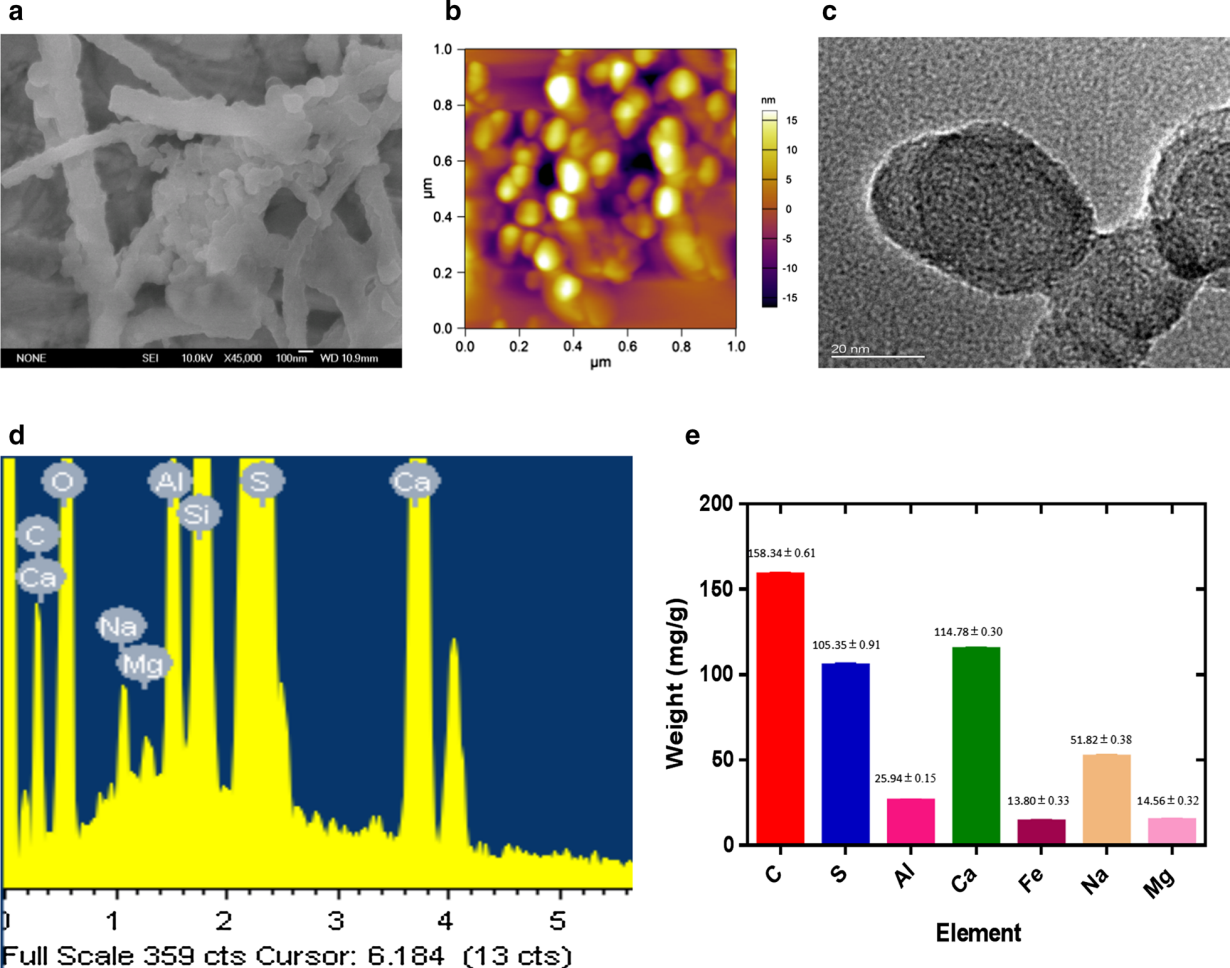
Physicochemical characterization of airborne nanoscale particles (ANP). **a** Scanning electron microscope image of ANP. **b** Atomic force microscopy image of ANP. **c** Transmission electron microscope image of ANP. **d** Chemical element analysis of ANP by EDS. **e** Quantitative element analysis of ANP by ICP-MS (metals) and element analyzer (carbon and sulfur)

Airborne nanoscale particles from collected indoor airborne pollution samples were examined. Firstly, SEM was applied to characterize the observed samples. SEM demonstrated that each observed nanoscale matters were aggregated by several smaller nanoscale particles (Fig. 5a). AFM/TEM observation further proved these findings. Before that, airborne samples were filtrated (< 1 μm) to remove larger size particles and fibers. The scanned area is randomly selected in AFM/TEM. Images showed that most of the nanoscale particles were in ten nanometres to one hundred nanometres (Fig. 5b, c). Based on EDS analysis, the ANP consist of carbon with smaller contributions of metals (Fig. 5d). We quantitative analyzed the chemical elements via ICP-MS (metals) and element analyzer (carbon and sulfur) (Fig. 5e).

### ANPs promote coagulation abnormality based on a 3D human microvascular model

**Fig. 6:**
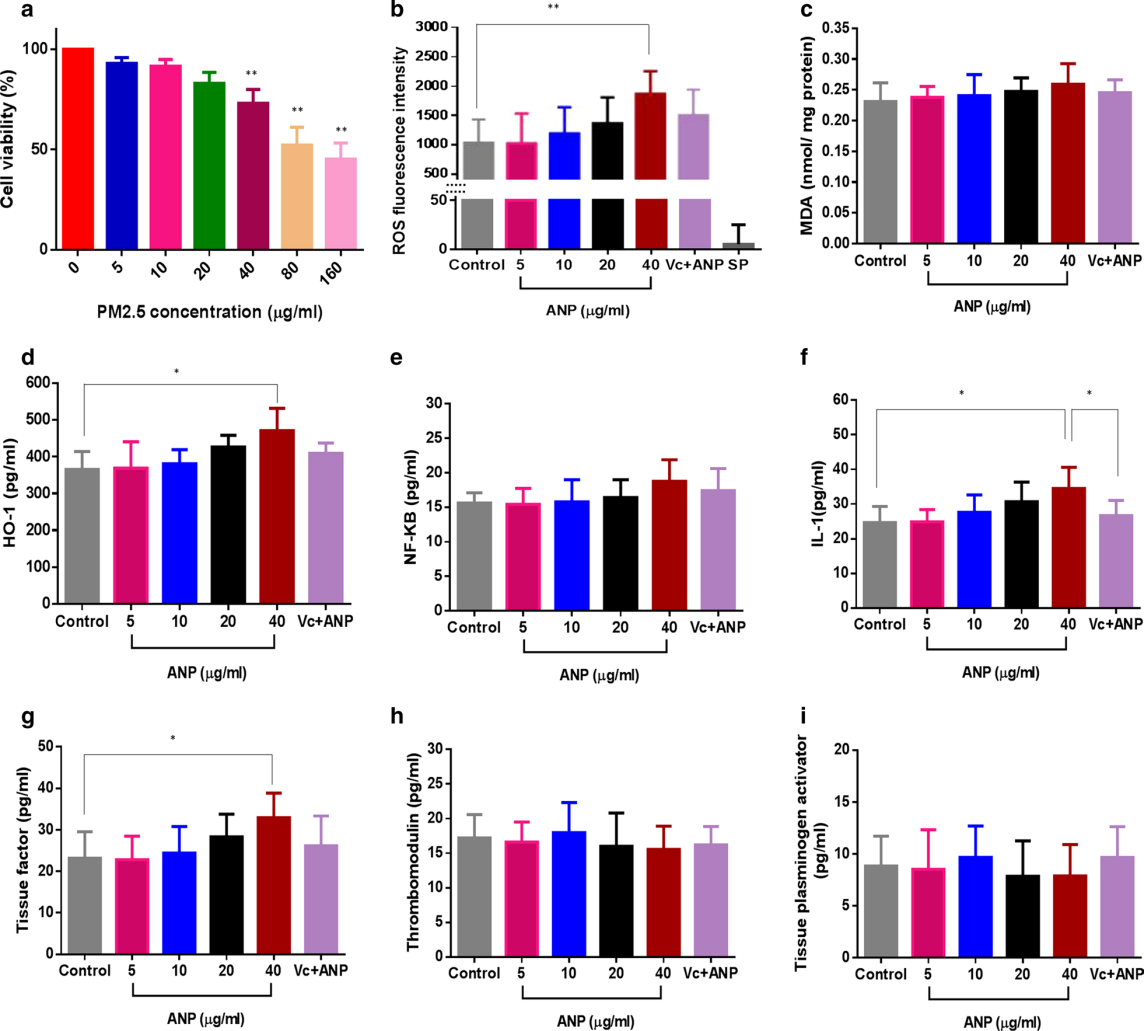
**a** Cell viability of HUVECs; ANP promotes coagulation abnormalities based on a 3D human microvascular model: **b** reactive oxygen species (ROS), **c** malondialdehyde (MDA), **d** heme oxygenase-1 (HO-1), **e** NF-κB, **f** interleukin 1 (IL-1), **g** tissue factor, **h** thrombomodulin, **i** tissue plasminogen activator [Vc + ANP: Vitamin C-0.1 mg/ml, ANP-40 μg/ml, SP: special control-ANP (40 μg/ml) but without cells] (*P < 0.05, **P < 0.01, compared with the control, n = 5)

A cytotoxicity assay was applied to evaluate cell viability after HUVECs exposed to ANPs (5, 10, 20, 40, 80 and 160 μg/ml). Results demonstrated that ANP possesses cytotoxicity and cell-inhibiting effect. HUVEC cytotoxicity was positively associated with ANP concentrations (Fig. 6a). High doses (80 and 160 μg/ml) induced significant cytotoxicity of HUVECs (cell viability < 50%)(Fig. 6a). Based on the established human microvessels, 3D cultured HUVECs were exposed to ANPs with concentrations of 5, 10, 20 and 40 μg/ml. This model can be used to simulate the physiological response of human vessels to ANP stimuli. Based on results from animal experiments, endothelial dysfunction is often accompanied by ROS up-regulation. In this research, we used fluorescent agents to identify the cellular expression of ROS after ANP stimulation for 24 h. By comparing the ROS generation of control and particles, the intracellular ROS was increased with increasing ANP concentrations. 40 μg/ml of ANP resulted in an apparent ROS increase (P < 0.01). However, administering an antioxidant (vitamin C) was effectively repressed ROS generation (Fig. 6b). ROS are key signaling molecules that play an essential role in the progression of oxidative stress and inflammatory disorders. Our results suggest that ANP could stimulate the human vascular endothelial cell to generate ROS which may further provoke lipid peroxidation and inflammatory response. It was expressed in MDA accumulation and up-regulation of HO-1, NF-κB, and IL-1 (Fig. 6c–f). Moreover, the higher ANP group (40 μg/ml) induced an increment of MDA, HO-1, NF-κB and IL-1 (Fig. 6c–f). However, an antioxidant (vitamin C) effectively suppressed the inflammatory responses resulting from ANP stimulation. All the results demonstrated that ANP is expected to activate human vascular endothelial cells via ROS induced oxidative stress.

Activation of vascular endothelial cells has been proven to induce endothelial cell dysfunction via animal models. In this research, we studied the relationships between ANP stimulation, procoagulant effects, and endothelial cell dysfunction in 3D human microvessels. These important biomarkers were measured after ANP stimulation of the 3D human microvessels. Tissue factor (TF) is the primary initiator of the coagulation cascade. We measured the TF content after ANP exposure to 3D human microvessels. Our results demonstrated that ANP stimulated vascular endothelial cells to express TF. The abnormal expression of TF was positively correlated with the ANP exposure concentration (Fig. 6g). Moreover, ANP affected the regular expression of TF as compared to the control group (Fig. 6g). Meanwhile, Vitamin C effectively repressed the secretion of TF by ANP stimulation (Fig. 6g). In addition to TF, we also measured TM and t-PA. Thrombomodulin (TM) is an endothelial cell receptor for thrombin. It functions as a natural anticoagulant by greatly accelerating the activation of protein C by thrombin. Tissue plasminogen activator [t-PA] is another protein found on endothelial cells that is involved in the breakdown of coagulation. Our results demonstrate that ANPs induced a disordered expression of TM (Fig. 6h) and t-PA (Fig. 6i). In summary, our results indicated that ANP is expected to activate endothelial cells via ROS generation. This is manifested in trigger upregulation of pro-inflammatory cytokines, as well as the disorder introduced into the coagulation and fibrinolysis systems.

### Biological interaction of ANP and protein

**Fig. 7:**
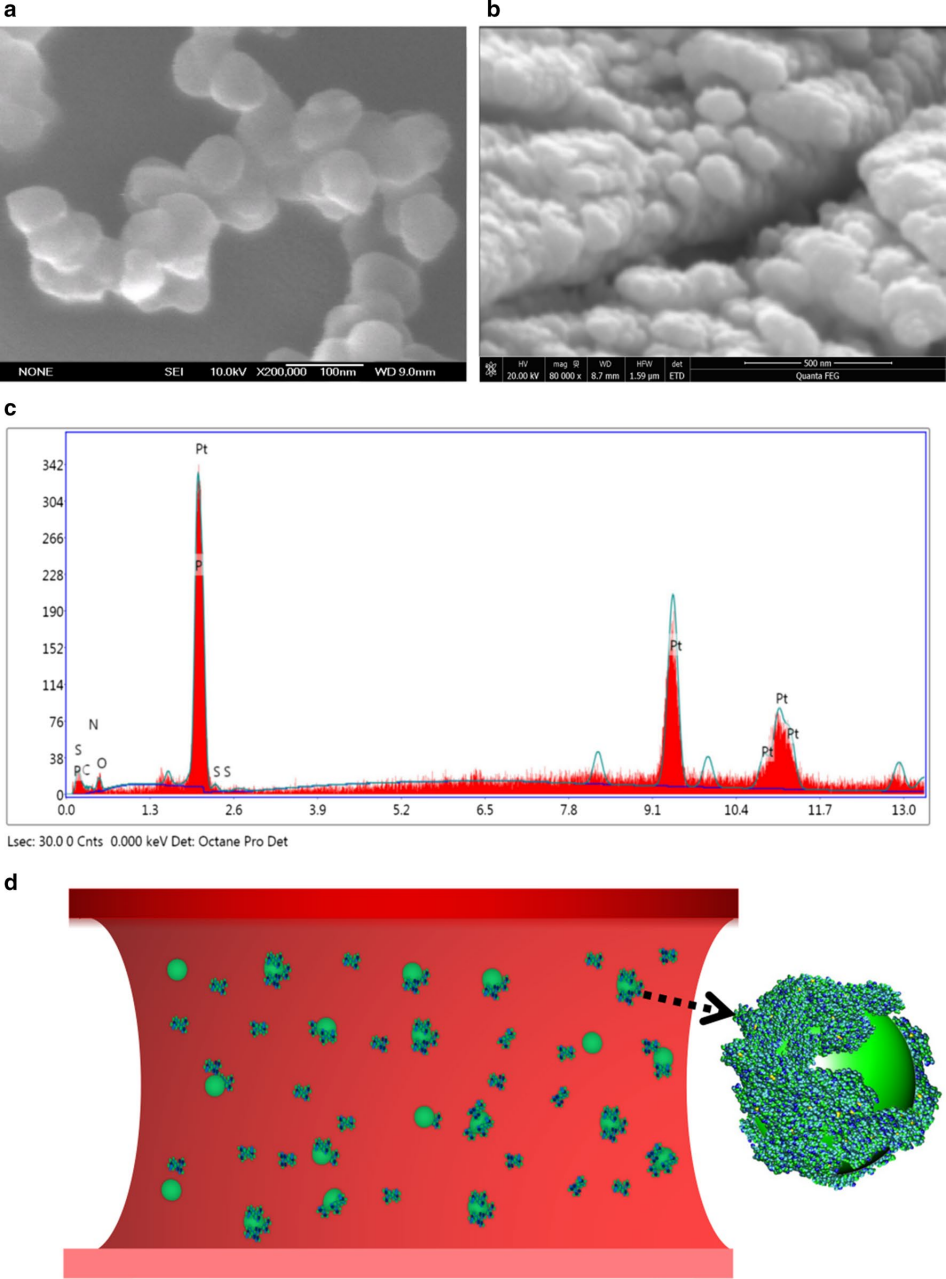
Characterization of ANP-plasma protein corona. **a**, **b** SEM images of ANP and ANP-protein corona. **c** Chemical elements analysis of protein corona by EDS. **d** The simulation diagram of ANP interaction with proteins in a human blood vessel

We studied the potential bio-interaction between ANPs and plasma proteins in this research. SEM demonstrated that the naked ANP particle was spherical or roughly spherical (Fig. 7a). Every naked ANP was less than 100 hundred nanometers. However, after ANPs were incubated with plasma protein, SEM demonstrated that the observed “matter” was larger than the original diameter of the naked ANP particle (Fig. 7b). All of the observed “matter” had an irregular morphology. The increase in dimension compared to the average diameter of a naked ANP particle can be attributed to the “protein coat”. Chemical element analysis of the “coat” was also performed to verify the existence of protein corona. EDS result demonstrated the coat was contained main elements of protein (C, O, N, S, P) (Fig. 7c). All these results suggest that the “protein corona” is formed after ANP interacted with the proteins. The formation of the “protein corona” proves that there is the potential bio-interaction between ANP and plasma protein (Fig. 7d).

### Biological interference of ANP with HS

**Fig. 8:**
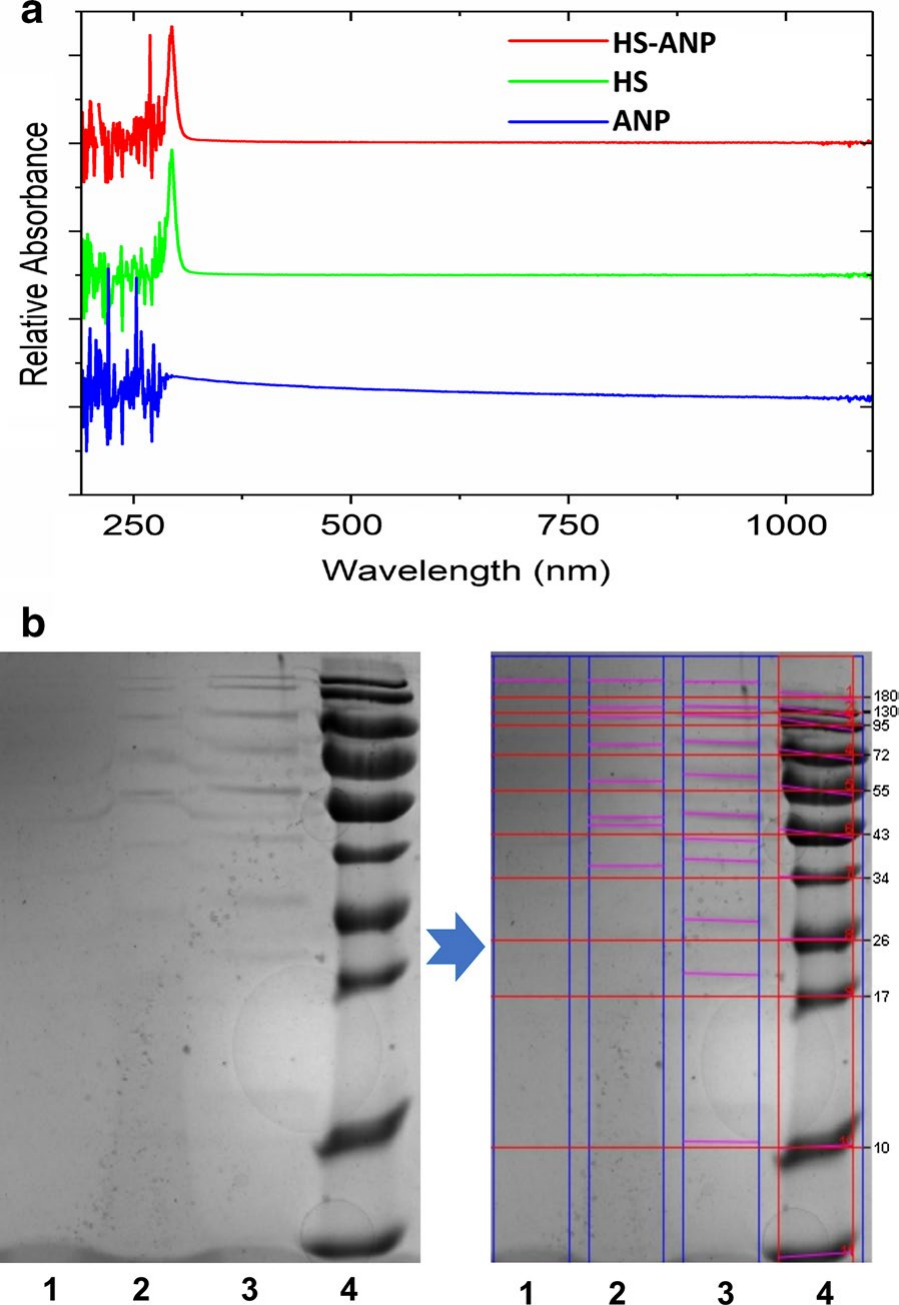
Physicochemical characterization of the biointeraction between ANP and HS. **a** UV–Vis spectrum of ANP, HS, and ANP–HS. **b** The SDS-PAGE analysis of ANP, HS, and ANP–HS. (1: ANP, 2: ANP–HS, 3: HS; 4: Ladder). Red lines marked protein bands

In this study, we have proved that proteins adsorb on the ANP surface. It is therefore essential to understand the connection between HS adsorbed on the ANP surface and subsequent biological outcomes. First, UV–Vis spectrophotometry was used to analyze naked ANP and HS-conjugated ANP. HS exhibits a sharp absorption peak, which is similar to HS-conjugated ANP (Fig. 8a). These absorption peaks are different from that obtained for pure ANPs. The UV–Vis spectrophotometry results show that ANP absorbs HS via bio-interaction. In addition, sodium dodecyl sulfate-polyacrylamide gel electrophoresis (SDS-PAGE) was examined to reveal the biological outcomes as ANP absorbs HS. Figure 8b shows the SDS-PAGE of HS-coated ANPs compared to ANPs after incubation for 24 h. HS sample exhibits a significant difference with heparan sulfate-conjugated ANP.

## Discussion

Research has shown that inhalation of particulate pollution can have adverse health impacts [41, 42]. PM2.5 has a most significant adverse impact on public health [43]. A well-characterization of selected model particles is meaningful to toxicological research of particle [44]. Based on SEM/AFM/TEM, we observed that ANPs are present in indoor PM2.5 samples. PM2.5 is a mixture of tiny particles with complex chemical composition. Based on elemental analysis (ICP-MS and element analyzer), APNs consist of carbon with smaller contributions of metals. Researches already reported the potential toxicity of ultrafine carbon particles contain metals. ROS generation and oxidative stress is still the best-developed paradigm to explain the toxic effects [45]. ANPs are more likely than larger particles, to be produced by fuel combustion [46], and ANP can remain in the atmosphere for weeks as compared to larger particles [47]. Environmental monitoring results have shown that indoor concentrations of fine particles are often higher than those typically encountered outdoors. Therefore, we should pay more attention to indoor ANPs since people spend most of their time in various indoor environments.

Numerous epidemiological studies indicate that ambient particulate matter in air pollution is strongly associated with increased cardiovascular disease. Based on animal models, the latest nano research shows that nanoscale could facilitate the migration of nanoparticles across any biological barrier [48]. Once inhaled, ANP can more easily cross the lung-blood barrier than the micron-sized PM2.5 [49]. Researchers speculate that ANPs play a vital role in the disruption of human vascular function [50]. However, we need to recognize that there are potential differences between an animal model and humans. It is impossible to extrapolate the hazards of ANP exposure from a simple animal model to the human cardiovascular system. The failure to translate from animals to humans is likely due to poor homology [51]. To better narrow the species difference, we utilized a 3D organotypic human microvascular network. This 3D vascular model maintains a physical structure which can closely mimic the biofunctions of human vessels. This model may provide a bridge for how in vitro outcomes translate to human responses.

A 3D HUVEC culture is distinct from a 2D culture. In this research, we compared a 2D HUVEC culture with a 3D HUVEC culture. In the 2D culture, we observed that the HUVECs adhered and spread on the lower plastic surface. Cell to cell contact in a monolayer is remarkably distinct from the vascular morphology in true in vivo situations. As compare to 2D culture, the established 3D microvascular model provided a more reliable human physiological response to ANP stimulation. Furthermore, the fibrin hydrogel has a sensitive response to external environmental stimuli [52–54], and enables us to study changes in the cell microenvironment or cell behaviors in a 3D model after ANP stimulation. In addition to being able to generate vessel-like structures, this model can also simulate blood fluidity with microfluidic technology.

In this research, a microfluidic system was integrated with a 3D cell culture to generate an organ-on-chip model. This model is a valuable tool to increase the physiological relevance of the 3D microvascular networks. Based on this chip, the flow force could be generated and regulated by a difference in liquid levels in two media channels. Fluorescent microparticles (FM) were initially loaded into any two reservoirs connected to the same media channel. With the aid of fluid flow, the microfluidic device provides a mean to enables the FM to go through the artificial human vessels. The microvessel exhibited good perfusion ability based on tracking the FM movement. Fluidity is one of the crucial aspects of vessels. Blood flow ensures continuous nutrition delivery, oxygen supply, and waste removal facilitated by blood pressure [55]. The flow in the microvessel chip can capture transport phenomena in real human vessels [56]. This dynamic microvessel model makes it possible to administer ANPs in 3D human microvascular networks. This method of exposure is useful in simulating the dynamic motion of ANP within human vessels. Because the dynamic exposure path is more realistic as compared to depositing ANP on a HUVEC monolayer (2D culture).

Studies have consistently shown that exposure to the fine particle is conducive to thrombus formation in humans [57, 58]. It is regarded as a trigger for cardiovascular disease, although the mechanism is unclear. A series of research indicate that the leading cause is directed to the endothelial cell damage or platelet activation. For example, Zhou et al. reported that fine airborne particles could induce oxidative damage of vascular endothelial cells [59]. Bihari et al. revealed that carbon nanomaterials could activate platelets after incubated with citrated blood in vitro [60]. Based on these experimental dose (fine airborne particles: 5 to 200 μg/ml [59]; carbon nanomaterials: 1 to 100 μg/ml [60]) in vitro, we measured the cytotoxicity of HUVECs after exposed to different ANPs (5, 10, 20, 40, 80 and 160 μg/ml). Cell viabilities are deceased with the increase in ANP concentration. Moreover, high doses (80 and 160 μg/ml) induced significant cytotoxicity of HUVECs (cell viability < 50%). Cells with extremely low viability are unsuitable for measurement of cellular bioreaction via xenobiotic stimulation. Besides that, low viability also leads to cell hardly extension in hydrogels. Given the above, ANPs (5, 10, 20, and 40 μg/ml) were loaded in 3D HUVEC vascular model to study procoagulant mechanism.

A certain content of ANP exists in PM2.5. ANP has a considerable surface area that may play a role in the pathogenesis of thrombus formation. In this research, we loaded ANPs into the dynamic 3D human microvascular network. The replicated 3D human vessels can be created to mimic the complex and dynamic 3D network present in real human blood systems. The primary aim of this study was to investigate any potential links between ANP exposure and coagulation abnormality. Following ANP exposure, we first observed the preliminary bioreaction of the 3D human microvessels by ROS measurement. As natural by-products of aerobic metabolism, ROS is always generated in response to stimulation by cytokines or xenobiotics [61]. Many studies have demonstrated that ROS regulate cellular signaling pathways involved in pathological processes [62, 63]. Based on the model, we observed that ANP could stimulate ROS elevation in 3D human vessels. Administering an antioxidant (vitamin C) is seen to repress ROS generation effectively. Results have shown that ANP could provoke oxidative stress in human 3D microvessels. There is increasing evidence demonstrating that abnormal elevation of ROS is always associated with oxidative stress which is implicated in diseases. For example, oxidative stress is associated with endothelial dysfunction [64]. It also plays a critical role in late thrombosis [65]. Consequently, it is reasonable to assume that ANP stimulated oxidative stress is intimately involved in vascular dysregulation.

Endothelial dysfunction is a marker of coagulant function abnormality and contributes to the development of thrombosis. Oxidative stress has been implicated in the development of endothelial dysfunction by activating vascular endothelial cells [66]. In this research, after introduced into 3D human microvessels, ANP was shown to activate endothelial cells via ROS accumulation. The endothelial cell activation process is a dynamic process with lipid peroxidation and HO-1 release. HO-1 plays a crucial role in the preservation of tissue integrity against oxidative stress, which contributes to the modulation of inflammatory responses in vivo [67]. Accompanied by oxidative stress, an intermediate amount of ROS also triggered an inflammatory response through the activation of NF-κB. The classical NF-κB-activating pathway is induced by a variety of innate and adaptative inflammatory mediators, such as proinflammatory cytokines (IL-1) [68]. Besides that, IL-1 was also released from 3D human microvessels after ANP stimulated endothelial cells. Moreover, this finding was in a positive ANP dose-dependent manner. Endothelial cell activation is typically induced by pro-inflammatory cytokines (e.g. IL-1) [69]. Following our exploration of ANP induced ROS elevation, we observed differing degrees of increase in IL-1 levels in the 3D human microvessels. An antioxidant (vitamin C) effectively suppressed this increase in IL-1 resulting from ANP stimulation. These results suggest that endothelial dysfunction is probably associated with ANP induced oxidative stress. Only the higher dose of ANP (40 μg/ml) significantly increased IL-1 expression in comparison to the other ANP groups. In comparison to 2D cells, 3D micro-tissues may demonstrate an innate resistance to ANP stimulation. Many studies have already reported that results from 2D cells may have overestimated the toxic effects [70]. It is due to results from 2D cell cultures do not adequately represent the functions of 3D tissues [71]. 3D tissues have extensive cell–cell and cell–matrix interactions, which increases biological tolerances [72].

IL-1 is a potent inducer of procoagulant activity in the human vascular endothelium [73]. The endothelial procoagulant activation induced by IL-1 may represent a trigger for systemic clotting activation in human vessels. In this research, we observed that ANP stimulated abnormal expression of tissue factor (TF). TF is a cell surface glycoprotein responsible for initiating the extrinsic pathway of coagulation. Following endothelial activation, the TF:FVIIa complex activates the coagulation protease cascade, which leads to fibrin deposition and activation of platelets [74]. Based on the 3D vessel model, we found that vessels exposed to high levels of ANP could exhibit aberrant expressions of TF. Inhibition of TF would be expected to reduce thrombosis associated with cardiovascular disease. In this research, an antioxidant can effectively inhibit TF expression. These results suggest that ANPs were potentially interfered with the procoagulant pathway via activate oxidative stress. Thrombosis does not depend on the procoagulant system alone, but also on the anticoagulant system. In the human body, there is a dynamic equilibrium between coagulation (procoagulant) and fibrinolysis (anticoagulant) systems [75]. Dysregulation of pro- and anticoagulants contributes to the coagulation abnormality which could further develop into thrombosis [76, 77]. To explore the potential relationship between ANP and coagulation abnormality, we also measured the ANP impact on anticoagulants. In addition to the aberrant TF expression, our results showed that ANPs also induced disordered expression of TM and t-PA. TM and t-PA both play essential roles in the anticoagulant system. TM is an endothelial cell receptor, which plays an essential modulating role in the anticoagulant response after vascular injury. TM can directly block the procoagulant activities of thrombin such as fibrinogen clotting, Factor V activation, and platelet activation [78]. t-PA is another essential substance which is synthesized in endothelial cells and is involved in the breakdown of blood clots. t-PA catalyzes the conversion of plasminogen to plasmin (the primary enzyme responsible for clot breakdown) [79]. In 3D human vessels, ANPs inhibited the regular expression of TM and t-PA, which severely weakens the normal anticoagulant function. ANP stimulated inflammatory cytokines (IL-1) are believed to activate the coagulation cascade and inhibit fibrinolysis. Such changes accelerate procoagulant responses which initiate the pathogenic development of thrombus formation.

Protein-nanoparticle interactions have been reported to induce changes in protein structure [80, 81]. Compared to micron-sized indoor particles, ANPs are believed to exhibit some unique physicochemical properties. Due to a larger proportion of surface atoms, ANPs have higher surface energy than larger sized particles. The higher surface energy enables ANPs to easily attract biomolecules on their surface [82–84]. Therefore, it is reasonable to speculate that a protein layer may envelop ANP once they come in contact with intravascular macromolecules. Plasma protein is extremely associated with coagulation [85, 86]. In this research, after binding with plasma protein, ANPs could be surrounded by a protein cloud to form a “protein corona”. Our findings suggest that ANPs may interfere with coagulant by interacting with plasma proteins. It conforms to previous research of nanoparticle could interact with plasma proteins to form “protein corona” [87]. Lundqvist et al. revealed the physiological mechanism of hematological toxicity by nanoparticle (NP) interacted with plasma protein. Exposure of foreign NPs to blood can result in the adsorption of protein layers that often triggers activation of the immune system (e.g. complement activation) [88]. For example, fibrinogen-absorbed NPs can trigger platelet adhesion and initiate thrombogenesis. Meanwhile, fibrinogen-absorbed NPs also can trigger hemostasis via a well-known regulator of the inflammatory response. This includes stimulate the NF-κB signaling pathway and lead to the release of inflammatory cytokines. These findings are consistent with the result of this research [89].

Heparan sulfate (HS) is one of the main components of vascular endothelial glycocalyx. It presents on the surface of endothelial cells. HS plays a physiological role in regulating levels of anticoagulant activity within the blood vessel [90]. HS achieves its anticoagulant activity by interacting with antithrombin, which then undergoes a conformation change, with the generation of the active form of antithrombin that inhibits blood coagulation factors [91]. In this research, based on UV–Vis and SDS-PAGE, ANPs are shown to changed HS fundamental structure via biological interaction. It indicated that HS structure could change upon binding to the ANP surface. Given these, our results suggest that ANP–HS interaction could potentially influence the protein function. It may weaken HS binding ability and accelerate procoagulant responses in blood [92]. However, airborne particles involved anticoagulant event is entirely unclear. Therefore, the procoagulant effect of ANP in the actual state with physiological concentration is to be studied further. It is useful to simulate the pathogenesis of ANP induced coagulation abnormality.

## Conclusion

This study is the first time, to our knowledge, that a 3D organ-on-chip model has been used to evaluate the toxicity of indoor air pollution on human vascular networks. Based on our results, we concluded that ANP does not only disrupt the normal coagulation function but also directly act on anticoagulant factors. These experimental observations provide a plausible biological explanation for the epidemiologically established link between ANPs and coagulation abnormality. This 3D human microvessel model provides a simple and versatile platform to study human vascular toxicity of PM2.5. It can provide more physiological characteristics than that obtained from 2D cell culture. However, the organ-on-chip is still unable to simulate all the physiological response and characteristics. With the combination of animal test, especially in real human respiratory concentrations, a more comprehensive mechanism study is to be done later.

## Methods

### ANP collection and characterization

The sampling site was located in Wuhan which is the largest industrial city in Central China. The population of Wuhan is 10.9 million people, and there are approximately 3 million vehicles. Indoor airborne particles were collected from nonsmoking homes that were distant from primary sources of particulate matter. During monitoring, smoking tobacco and cooking were not allowed in the rooms. The indoor airborne particles were collected on quartz filters using a high-volume PM2.5 sampler (Anderson, USA). Each quartz filter was cut into smaller pieces of about 2 cm^2^. The cut pieces were put into a sterilized beaker with 90 ml sterilized pure water. A 20 min sonification was carried out at temperatures lower than 20 °C. Airborne nanoscale particles were filtrated (< 1 μm) to remove larger size particles and fibers. The collected suspensions were freeze-dried in a vacuum for 24 h, and stored at − 20 °C. The morphology and size of the collected indoor air particles were observed using AFM (SPM 3100, Veeco Instruments, Inc., USA), SEM (JSM-6700F, JEOL) at 10 kV and TEM (JEOL JEM-2010F) at 200 kV. Elements of ANPs were analyzed by using SEM–EDS, ICP-MS and element analyzer.

### Cell culture and gel precursor preparation

GFP-HUVEC (Neuromics, USA) or Non-GFP-HUVEC (Lonza, USA) were cultured up to passage 6 in endothelial growth medium (EGM-2, Lonza). HLFs were cultured up to passage 10 in fibroblast growth medium (FGM-2, Lonza). Cell cultures were grown to 80% confluence before passage and use in experiments. DPBS (Gibco, USA) was preheated at 37 °C for 2 h. The fibrinogen solution (15 mg/ml) was prepared by dissolving 15 mg bovine fibrinogen (Sigma, USA) in the preheated DPBS. Thrombin (100U, Sigma, USA) solution was dissolved in 1 ml DPBS which contained 0.1% BSA.

### Microvascular network formation

PBS, Trypsin–EDTA and endothelial cell growth medium were preheated in the 37 °C water bath. The media were removed and discarded from the cell flask, and the cells then rinsed with PBS. 1 ml of Trypsin–EDTA was added to the flask, mixed briefly, and incubated at 37 °C for a few minutes to release the cells. HUVECs were resuspended at a concentration of 12 million cells per ml. HLFs were resuspended at a concentration of 6 million cells per ml. 1 µl thrombin solution (100 U/ml) was mixed with 20 µl fibrinogen solution (15 mg/ml, Sigma, USA), 79 µl HUVECs (12 × 10^6^ cell/ml) and 20 µl HLFs (6 × 10^6^ cell/ml). Ten microliter of this mixture was immediately injected into the central gel channel of the microfluidic device. Devices were placed in a humidified incubator at 37 °C, and the cell mixture was allowed to gel for 40 min. The EGM-2 media were then loaded in the media channels, and the media were replaced every 24 h. The microfluidic device was cultured in a humidified incubator for 4 days at 37 °C and 5% CO_2_. During the progress of microvascular development, images of the 3D HUVEC networks were captured by a camera linked to a confocal microscope (Olympus IX81, USA). Images of the fluorescent micro-vessels were captured by a confocal microscope (Olympus IX81, USA).

### Perfusion measurement of microvascular networks

Perfusion of 3D cultured micro-vessels is almost exclusively the domain of microfluidic techniques. In this research, we measured the perfusion capability of the microvascular networks in the microfluidic device. Perfusion of the microvasculature was measured via microparticle flow (Invitrogen, USA) through the microvascular network consisting of continuous and perfusable lumens. Briefly, the microparticle solution was diluted 100-fold with EGM-2 medium. Fill 20 µl of this medium into any two syringe barrels that are connected to the same media channel. Fluid flow in a media channel creates a pressure drop along the channel. The movement of the microparticles within the generated perfusable lumens was captured using a confocal microscope (Olympus IX81, USA).

### Measurement of cell viability and ANP induced coagulation dysfunction

ANPs were collected by using a sterilized filter (< 1 μm) to remove larger particles as well as any fibers from the quartz. Particle exposure medium was prepared of ANP dissolved in the EGM-2 for 10 min sonification. A cytotoxicity assay was applied to evaluate HUVEC cell viability using Cell Counting Kit 8 (CCK-8) (Biosharp, Hefei, China). After exposing the cells to ANPs (5, 10, 20, 40, 80 and 160 μg/ml) for 24 h, 10 μl CCK8 reagent was added to each well. After incubating for 4 h at 37 °C, the absorbance was detected using a Microplate Reader (PerkinElmer Inc., Connecticut, USA) at 450 nm. The survival rate of the untreated cells was set as 100%. The experiment was repeated three times.

Exposure solutions of ANPs (5, 10, 20, 40 μg/ml) and ANP-Vitamin C (0.1 mg/ml-Vitamin C and 40 μg/ml-ANP) were prepared. Each of the exposure solutions was loaded into the microvascular networks for 24 h at 37 °C and 5% CO_2_. Measurement of ROS, MDA, HO-1, NF-κB, IL-1 and coagulation factors (TF, TM, and t-PA) were performed as described here. Collected the initial medium after 24 h incubation. Then the devices were washed three times with the EGM-2 medium. Collected initial medium and washings which are further for assessment of coagulation factors (TF:Shenzhen Jingmei, China; TM:Wuhan Huamei, China; t-PA: Beijing Baiaolaibo, China). MDA (Nanjing Jiancheng, China), HO-1 (Abcam, USA), NF-κB (Abnova, Germany) and IL-1 (Nanjing Jiancheng, China) were measured using biokits according to the manufacturer’s instructions. ROS was measured using oxidation-sensitive fluorescent DCFH-DA (Merck KGaA, Germany), which is a non-fluorescent compound that is freely taken up by cells and hydrolyzed by esterases to 2′,7′-dichlorodihydrofluorescein (DCFH). Non-GFP-HUVECs were co-cultured with HLFs, fibrinogen and thrombin, and the mixtures (50 μl) then loaded into 96 well-plate. The microvasculature that formed was used in ROS assessment. Fluorescence intensities (ROS) were detected using a fluorescence reader (FLx800; BioTek Instruments, Winooski, VT, USA).

### ANP-protein corona characterization

Blood samples were obtained from the healthy mice (Hubei Province Experimental Animal Center, China). Mice experiment was approved by Central China Normal University (Approval ID:CCNU-IACUC-2016-003). Plasma protein was extracted via the instruction of total plasma protein-extraction kit (Bestbio, Shanghai, China). ANPs and plasma protein were mixed, vortexed and placed in an incubator at 37 °C for 24 h. The mixtures were transferred to a new centrifuge tube and centrifuged at 15,000×*g* for 20 min at 4 °C. Supernatants were removed, and pellets dispersed in 1 ml of cold PBS. The collected sample was transferred to a new vial and centrifuged again to pelletize the particle-protein complexes. Morphology and size of the ANP-protein complexes were observed using a scanning electron microscope (SEM, JSM-6700F, JEOL) at an acceleration voltage of 10 kV. Chemical element analysis of protein corona was approved by EDS.

### Identification of an interaction between ANP and HS

Heparan sulfate was obtained from Glyco company (Glyco, Shenzhen, China). Heparan sulfate-contained ANPs were analyzed by UV–Vis spectroscopy and SDS-PAGE. Preparation of Heparin-contained ANPs uses the same method described in the section of ANP-Protein corona characterization. For UV–Vis analysis, the absorption analysis was carried out from 200 to 1250 nm using ultraviolet–visible spectrophotometer (ThermoFisher, USA). For SDS-PAGE (gel electrophoresis kit, Beyotime^®^ Biotechnology, China), samples (ANP, Heparan sulfate, Heparan sulfate-ANP conjugate) were re-suspended in protein loading buffer [62.5 mM Tris–HCl pH 6.8, 2% (w/v) SDS, 10% glycerol, 0.04 M DTT and 0.01% (w/v) bromophenol blue] with a 4:1 ratio. Ten microliter of the boiled samples were loaded in different lanes of a 12% gel polyacrylamide gel. Gel electrophoresis were performed until the proteins almost reached the bottom of the gel. Subsequently, the gel was stained for about 1 h in coomassie blue stain [10% acetic acid, 50% methanol, 2.5% (w/v) brilliant blue] and washed overnight in 10% acetic acid and 50% methanol. Finally, the SDS-PAGE was scanned using a gel imaging system (ChemiDoc MP, Bio-Rad Laboratories, California, USA).

### Statistical analysis

GraphPad Prism software was used for statistical analysis of the experimental data, and for graphing the results. All data are presented as the mean and standard deviation (S.D.). Any statistical differences between groups were determined by ANOVA. The method of least significant difference (LSD) was used to compare the effects between each exposure group and the control. P < 0.05 and P < 0.01 were considered significant.

## Abbreviations

ANPs: airborne nanoscale particles
PM2.5: particulate matter (diameter ≤ 2.5 μm)
TF: tissue factor
TM: thrombomodulin
t-PA: tissue plasminogen activator
ROS: reactive oxygen species
IL-1: interleukin 1
UV–Vis: ultraviolet–visible spectroscopy
SEM: scanning electron microscope
AFM: atomic force microscope
EDS: energy-dispersive spectroscopy
GFP: green fluorescent protein
HLF: human lung fibroblasts
FBS: fetal bovine serum
DPBS: Dulbecco’s phosphate-buffered saline
HS: heparan sulfate
HUVEC: human umbilical vein endothelial cell
MDA: malondialdehyde
SDS-PAGE: sodium dodecyl sulfate-polyacrylamide gel electrophoresis
NF-κB: nuclear factor kappa B
HO-1: heme oxygenase-1
ICP-MS: inductively coupled plasma mass spectrometer
